# Association of PD-1 polymorphisms with the risk and prognosis of lung adenocarcinoma in the northeastern Chinese Han population

**DOI:** 10.1186/s12881-019-0914-8

**Published:** 2019-11-12

**Authors:** Kun Huang, Erqiang Hu, Wan Li, Junjie Lv, Yuehan He, Gui Deng, Jinling Xiao, Chengcheng Yang, Xinyu Zhao, Lina Chen, Xinyan Wang

**Affiliations:** 1 Department of Respiratory, the Second Affiliated Hospital, Harbin Medical University, Harbin, 150086 People’s Republic of China; 2 College of Bioinformatics Science and Technology, Harbin Medical University, Harbin, Heilongjiang Province China

**Keywords:** Lung adenocarcinoma staging, PD-1, Polymorphism, SNP

## Abstract

**Background:**

Lung cancer is a leading cause of death from cancer worldwide, especially non-small cell lung cancer (NSCLC). The marker of progression in lung adenocarcinoma, the main type of NSCLC, has been rarely studied. Programmed death 1 (PD-1) is an effective drug target for the treatment of NSCLC. Our study aimed to examine the PD-1 role in the disease process. The study of the effect of polymorphisms on the progression of lung adenocarcinoma in the Han population of Northeast China may provide a valuable reference for the research and application of these drugs.

**Methods:**

Chi-square test, Wilcoxon rank sum test, and classification efficiency assessment were used to test SNPs of PD-1 in 287 patients and combined with clinical information.

**Results:**

We successfully identified biomarkers (rs2227981, rs2227982, and rs3608432) that could distinguish between lung adenocarcinoma patients of early stages and late stages. Multiple clinical indicators showed significant differences among different SNPs and cancer stages. Furthermore, this gene was confirmed to effectively distinguish the stages of lung adenocarcinoma with RNA-seq data in TCGA.

**Conclusions:**

Out study indicated that the PD-1 gene and the SNPs on it could be used as markers for distinguishing lung adenocarcinoma staging in the Northeast Han population. Our investigation into the link between PD-1 polymorphisms and lung adenocarcinoma would help to provide guidance for the treatment and prognosis of lung adenocarcinoma.

## Background

Lung adenocarcinoma is the most common type of non-small cell lung cancer (NSCLC) and the leading cause of cancer death worldwide. It may develop as a result of the interaction between environmental risk factors and individual genetic susceptibility [1]. Therefore, genetic susceptibility to lung adenocarcinoma has become an area of interest in lung cancer research [9, 7]. Almost all lung adenocarcinoma patients were diagnosed in advanced stages (IIIB and IV) [4]. Lung adenocarcinoma develops silently and has no specific symptoms. Therefore, early lung cancer (Stage I and Stage II) is difficult to find. So accurate pathological staging is an important factor in the treatment of lung adenocarcinoma and plays an important role in the selection of postoperative treatment and prognosis. The Tumor, Node, Metastasis (TNM) staging system for NSCLC is an internationally accepted system used to characterize the extent of disease. Misdiagnosis still exists for patients with early hematogenous metastasis using TNM when imaging is not applicable.

Programmed death 1 (PD-1) plays a very important role in tumor immunity. PD-1 is currently the drug target of NSCLC [6]. Various studies have evaluated the relationship between PD-1 polymorphisms and various cancer risks. rs2227981 (PD-1.5) and rs36084323 (PD-1.1) were both related to NSCLC [14]. Previous studies reported the association between rs2227982 (PD-1.9) and gastric cardia adenocarcinoma [15] as well as breast cancer [12]. rs7421861 could increase the risk of colorectal cancer in Chinese [3]. Our study aimed to examine the effect of PD-1 and these four PD-1 polymorphisms on disease progression of lung adenocarcinoma.

In order to combine the lung adenocarcinoma staging information and clinical information, four polymorphisms of the PD-1 gene, including rs2227981 (PD-1.5), rs2227982 (PD-1.9), rs36084323 (PD-1.1) and rs7421861, were selected, and the genotypes of 287 patients were determined by SNaPshot primer extension assays.

## Methods

### Subjects

This was a cross-sectional study conducted among subjects comprised 111 healthy controls and 287 patients who were diagnosed with lung adenocarcinoma at the Department of Respiratory Medicine of the Second Affiliated Hospital of Harbin Medical University (Harbin, Heilongjiang, China) between 2013 and 2015. The patients were diagnosed by surgery and pathological assessment. The healthy control group was randomly selected from 111 age-matched healthy subjects without any history of familial or personal autoimmune diseases or malignancies, who received annual physical examinations at the same hospital. According to lung cancer classification developed by WHO in 2009, the histological classification and stage were assessed. These samples of patients were collected prior to treatment since different treatments may affect clinical indicator measures of patients. All patients and controls were recruited from the northeastern Chinese Han population. Table 1 lists the clinicopathological features of the patients and the controls. The written informed consent for blood collection and subsequent analysis was provided by each participant. The ethics committee of the same hospital approved the study.

**Table 1:** Clinical characteristics of case and control subjects

Variable	Case	Control	*p*-Value
Total	287	111	
Age(yr), mean ± SD	62.0 ± 10.1 (27–89)	62.5 ± 10.7 (40–91)	0.757
Gender			0.2535
Males	148 (52%)	65 (59%)	
Females	139 (48%)	46(41%)	
TNM^a^			
I-II	150 (12%)		
III-IV	137 (48%)		

^a^the 7th Edition of TNM in lung cancer, 2009

**Table 2:** Basic information of SNPs

rs	pos	Alleles	Functional consequence	*p*_value for Hardy-Weinberg equilibrium
rs2227981	2:241851121	C/G/T	synonymous codon, upstream variant 2 KB	0.84
rs2227982	2:241851281	C/T	missense, upstream variant 2 KB	0.19
rs36084323	2:241859444	A/G	upstream variant 2 KB	0.20
rs7421861	2:241853198	A/C/T	intron variant, nc transcript variant	0.81

Six clinical indicators, including carcino-embryonic antigen (CEA), neutrophilicgranulocyte (GRAN), lactate dehydrogenase (LDH), lymphocyte (LYM), Neutrophil to Lymphocyte Ratio (NLR) and white blood cell (WBC), were measured in all samples [2].

### DNA extraction and genotyping

The TIANamp Blood DNA Kit (TIANGEN, Beijing, China) was used to extract genomic DNA from 500 μl of EDTA-anticoagulated venous blood samples. Four SNPs of the PD-1 gene were genotyped: rs2227981, rs2227982, rs36084323 and rs7421861. Genotype was assayed by SNaPshot Multiplex Kit (PE Applied Biosystems, Warrington, UK and Foster City, CA, USA). Primer 3.0 was used to design the primers for use in PCR amplification. The primer sequences for each SNP were as follows:

- rs2227981: 5′-TCTCCTGAGGAAATGCGCTGAC-3′ (forward) and 5′-TGGTGTCCCCAGATCACACAGA-3′ (reverse);
- rs2227982: 5′-TCTCCTGAGGAAATGCGCTGAC-3′ (forward) and 5′-TGGTGTCCCCAGATCACACAGA-3′ (reverse);
- rs36084323: 5′-CTCCCATTCTGTCGGAGCCTCT-3′ (forward) and 5′-GAAGGGGAGGTCAGCCTCACAG-3′ (reverse);
- rs7421861: 5′-CCCAGCTGGAATGTCATTGAGAA-3′ (forward) and 5′-TTACACTCCCCTGTGCCAGAGC-3′ (reverse).

PCR was performed with 1 μL of DNA sample, 3.0 mM Mg^2+^, 1× GC-I buffer (Tahara), 1 μL multiple PCR primers, 0.3 mM dNTP and 1 unit HotStarTaq polymerase (Qiagen, Inc.) in a total volume of 20 μL. The PCR cycling program was as follows: 95 °C for 2 min; followed by 11 cycles of 94 °C for 20 s, 65 °C (decreased 0.5 °C per cycle) for 40 s and 72 °C for 90 s; plus 24 cycles of 94 °C for 20 s, 59 °C for 30 s and 72 °C for 90 s; with a final extension at 72 °C for 2 min and 4 °C forever. Next, 5 units shrimp alkaline phosphatase and 2 units Exonuclease I was added to the PCR product, incubated at 37 °C for 1 h and inactivated at 75 °C for 15 min for purification. SNaPshot multiple single base extension reaction was performed using 5 μL SNaPshot Multiplex Kit (Applied Biosystems), 0.5 μL 5′ ligase primer mixture (1.2 μM), 0.5 μL 3′ ligase primer mixture (1.6 μM), 2 μL ddH_2_O and 2 μL purified PCR product in a final volume of 10 μL. The reactions were cycled as follows: 28 cycles of 96 °C for 10 s, 55 °C for 5 s and 60 °C for 30 s, with the products subsequently kept at 4 °C. Purified extension product 0.5 μL was then combined with 0.5 μL Liz120 Size Standard and 9 μL Hi-Di, inactivated at 95 °C for 5 min and then sequenced and analyzed using an ABI 3730XL DNA Analyzer and GeneMapper 4.1 (Applied Biosystems Co.Ltd.USA), and the nucleotide at each SNP site was identified and recorded.

### Statistical analysis

#### Hardy-Weinberg equilibrium

The statistical test of Hardy-Weinberg equilibrium has been an important tool for detecting genotyping errors in the past and is still important in the quality control of next-generation sequence data. The chi-square test was used to test whether the 4 SNPs were in Hardy–Weinberg equilibrium with Excel.

#### Chi-square test

The chi-square test gives evidence of association or no association. The difference between the observed frequency and the expected frequency can be assessed by a statistical test called *χ*^2^ [10]. The statistical formula for this test is as follows:

$$ {\chi}^2=\sum \frac{{\left(O-E\right)}^2}{E} $$

where *O* is the observed cell frequency, and *E* is the expected cell frequency. The *P* value of the test was calculated by R program.

#### Wilcoxon rank sum test

The Wilcoxon rank sum test is a method often used in statistical practice to compare position measurements where the underlying distribution is far from normal or not known in advance [13]. We used the Wilcoxon rank sum test to analyze differences of the six indicators between different TNM stages. Then, for the early stage (TNM I and TNM II) and late stage (TNM III and TNM IV) of both- or single-gender samples, differences of six clinical indicators were also tested, separately.

#### Logistic regression analyses

Correlations between lung adenocarcinoma stages and SNP genotypes were analyzed by logistic regression model under dominant and recessive models. Logistic regression analyses were performed using IBM SPSS (Statistical Package for the Social Sciences) Statistics ver. 17.0.

#### Classification efficacy assessment

To evaluate the efficiency of classifying early and late stage samples of genes and clinical indicator-encoding genes, a Support Vector Machine (SVM) classifier was constructed. Leave-one-out cross-validation (LOOCV) was carried out to assess the performance. The receiver operating characteristic (ROC) curves were plotted and the areas under the curves (AUC) were computed. Those with better classification efficiency indicated their better potential to act as biomarkers.

## Results

### Differences of six indicators between different TNM stages

**Fig. 1:**
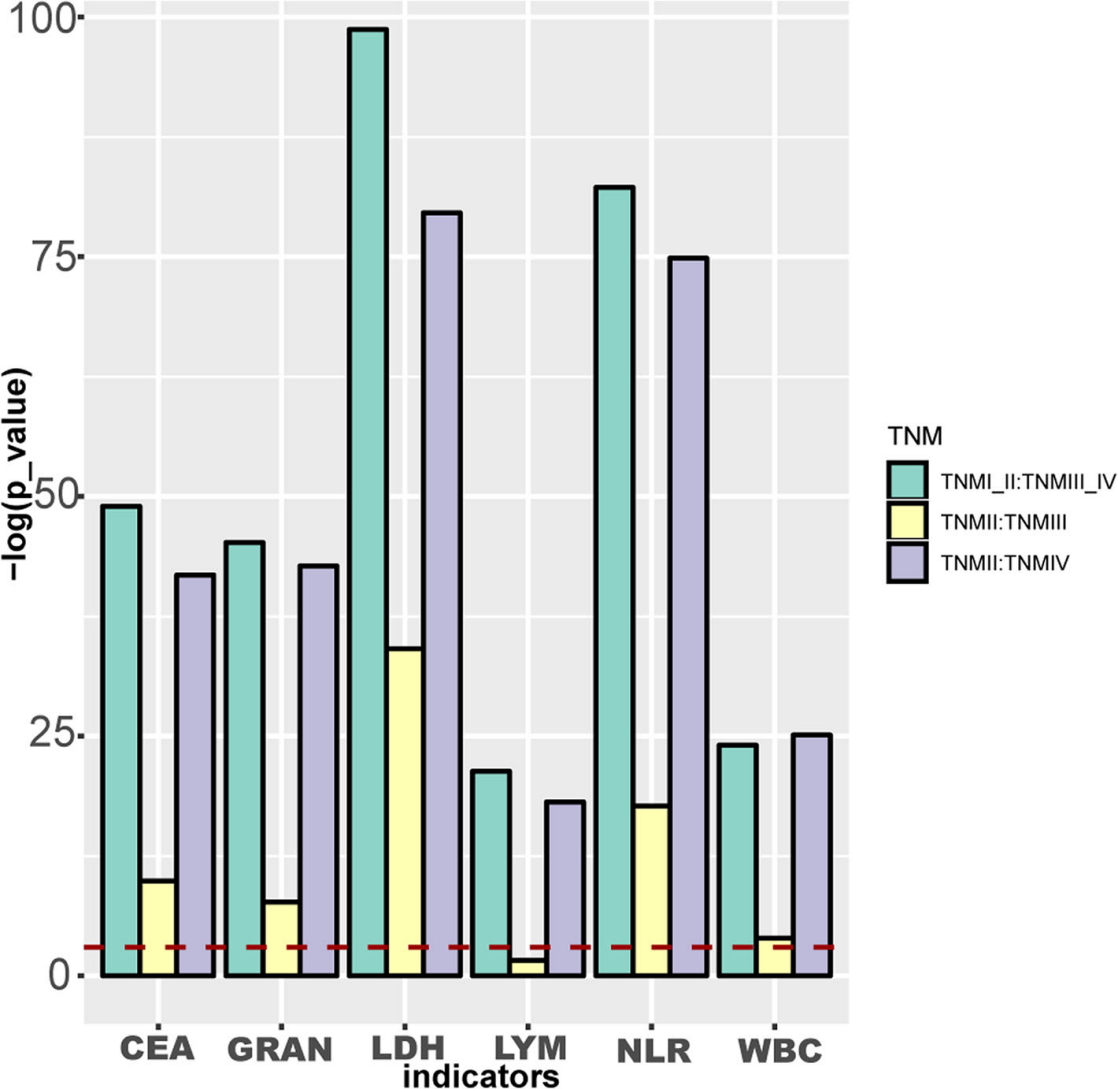
Association of TNM stages and six clinical indicators. The higher the column in the graph, the more significant it is. The red line represents the threshold of *p*-value = 0.05

For the six clinical indicators related to lung adenocarcinoma, the TNM staging of the patients was investigated, and the Wilcoxon rank sum test was used to obtain the significant *p* values for differences of the indicators at different stages (Fig. 1). The results indicated that there were significant differences in the 6 indicators between the early stage (TNM I and TNM II) and the late stage (TNM III and TNM IV). The difference between early and late stages was better than that between other stages. So the subsequent analysis was carried out in two stages.

### Association of PD-1 SNPs with lung adenocarcinoma in different models

**Fig. 2:**
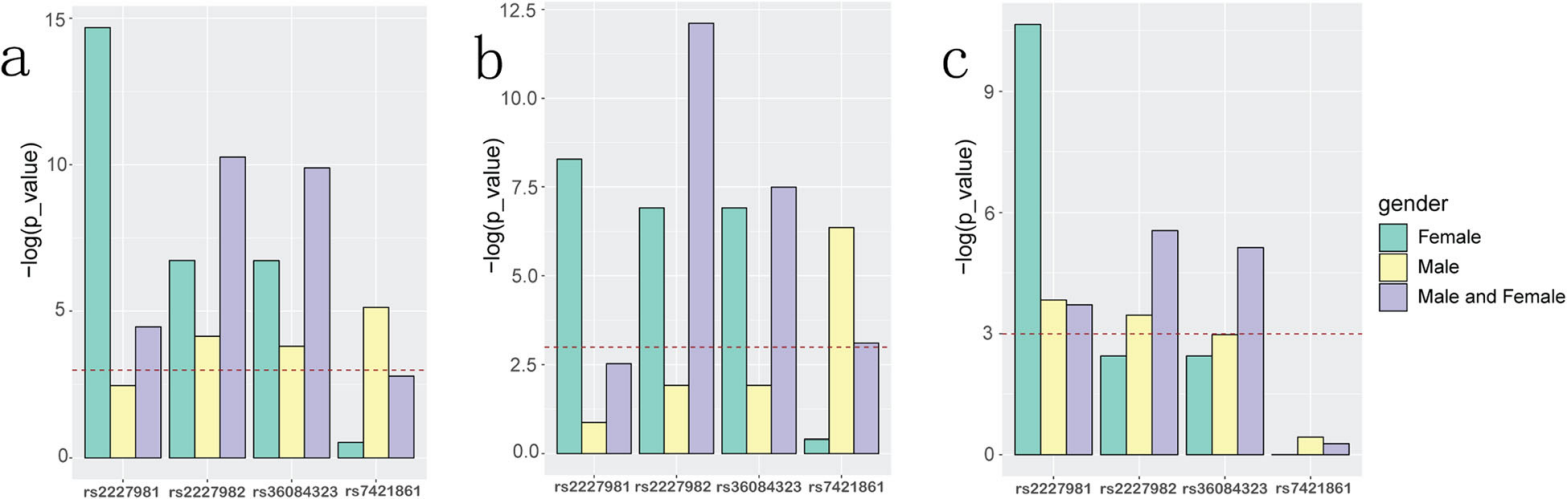
Association of PD-1 SNPs with lung adenocarcinoma stages in different models. **a** allele model; **b** dominant model; **c** recessive model. The dashed red lines indicate the significance threshold (*p*_value = 0.05) using chi-square test

For the four SNPs (rs2227981, rs2227982, rs3608432, rs7421861), we used chi-square test of three models (allele model, dominant model, recessive model) to test the association of SNPs with lung adenocarcinoma in all samples and single-gender samples, separately (Fig. 2). These results demonstrated that rs2227981 was significantly correlated with lung adenocarcinoma stages in allele model and recessive model. The women samples were significantly correlated with lung adenocarcinoma stages in all three models. SNPs rs2227982 and rs36084323 were significantly correlated with lung adenocarcinoma staging in all three models. The rs7421861 in male samples were significantly correlated with lung adenocarcinoma stages in allele model and dominant model. Therefore, rs2227981, rs2227982, rs3608432, and rs7421861 were expected to be markers to distinguish lung adenocarcinoma stages.

**Fig. 3:**
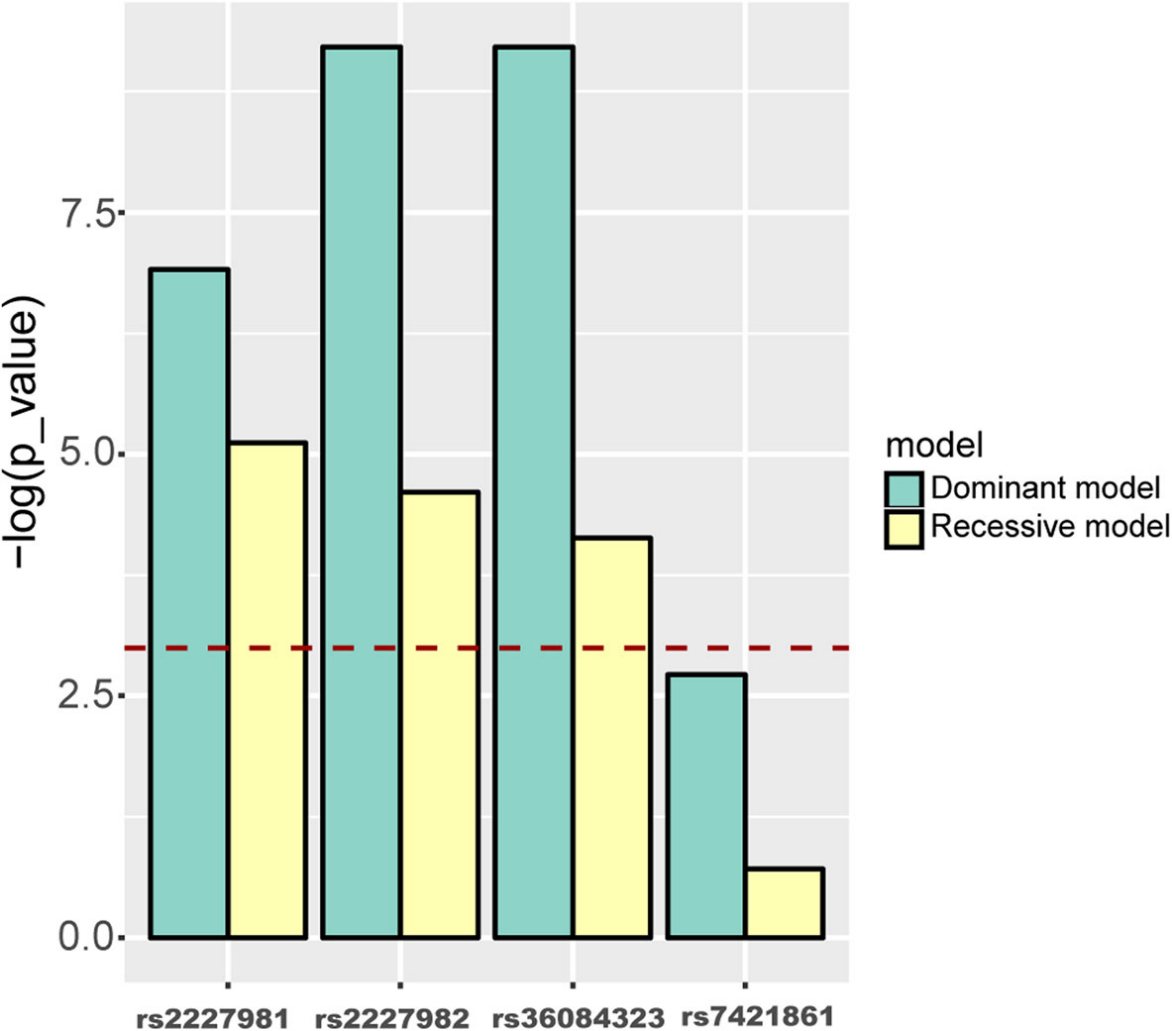
*P*_value of logistic regression model under dominant and recessive models. The dashed red line indicates the significance threshold (*p*_value = 0.05)

Furthermore, the correlation between lung adenocarcinoma stages and SNP genotypes were analyzed under dominant and recessive models, respectively (Fig. 3). Except rs7421861, the other three SNPs were significantly correlated with the stages of lung adenocarcinoma in both models.

In addition, we also used the Haploview software to test the correlation between SNP haplotypes and lung adenocarcinoma stages (Tables 3 and 4). Of the four haplotypes, three showed a significant correlation with lung adenocarcinoma stages. This further indicated that the four SNPs on PD-1 can be used as potential markers for lung adenocarcinoma staging.

**Table 3:** Association of single SNPs with lung adenocarcinoma

Name	Assoc allele	Frequency of early stage samples	Frequency of late stage samples	Chi square	*P* value
rs2227981	C	0.796	0.700	6.904	0.0086
rs2227982	T	0.507	0.333	17.835	2.41E-05
rs7421861	T	0.803	0.733	3.875	0.049
rs36084323	A	0.504	0.333	17.115	3.52E-05

**Table 4:** Association of haplotypes with lung adenocarcinoma

Haplotype	Frequency	Frequency of cases	Frequency of controls	Chi square	*P* Value
CTTA	0.413	0.500	0.333	16.41	5.10E-05
TCTG	0.253	0.201	0.300	7.475	0.0063
CCCG	0.233	0.197	0.267	3.875	0.049
CCTG	0.096	0.091	0.100	0.127	0.7218

### The difference of six clinical indicators in different stages and different SNP genotyping

**Fig. 4:**
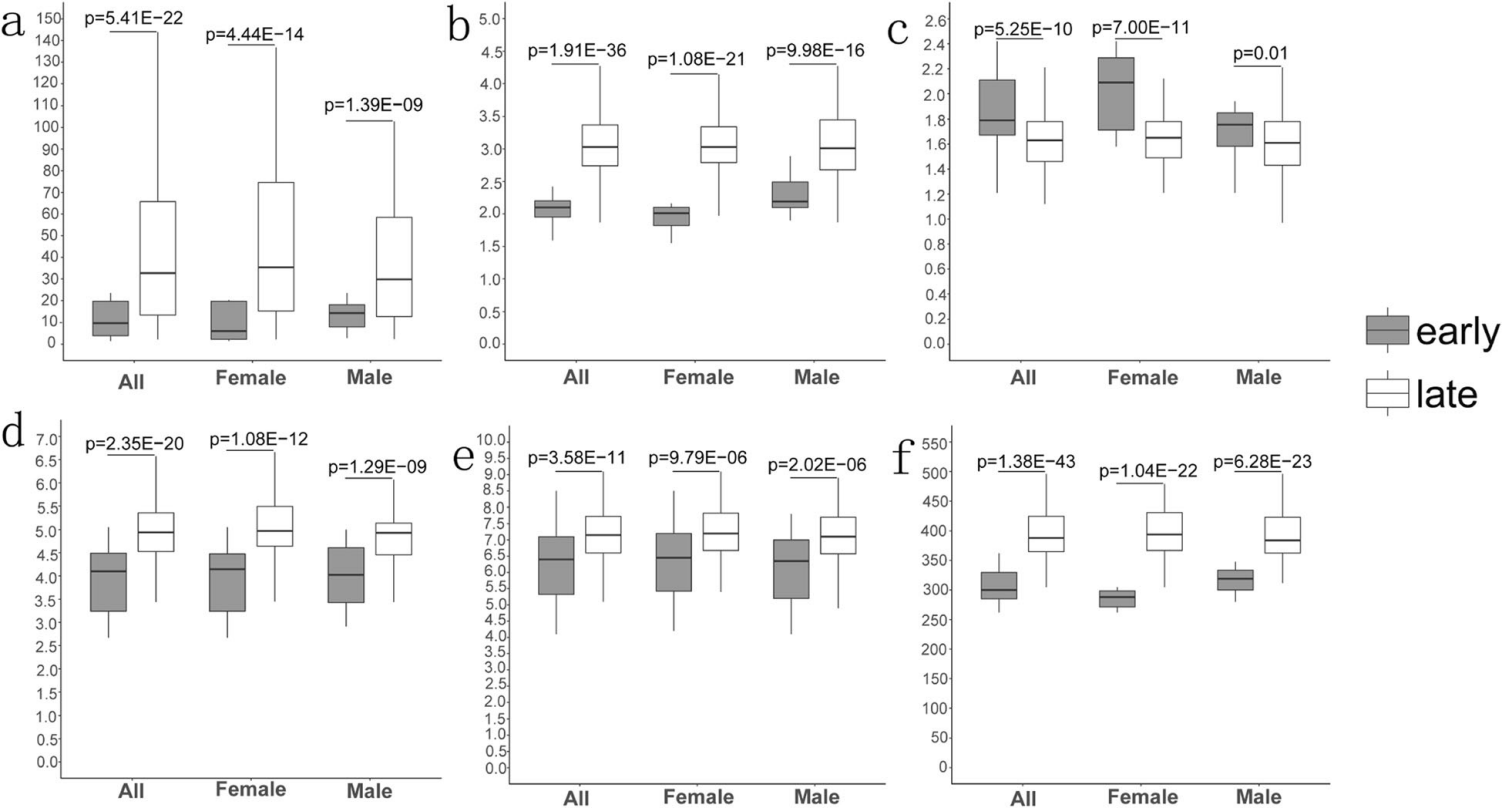
Difference of six clinical indicators in different stages. **a** CEA; **b** NLR; **c** LYM; **d** GRAN; **e** WBC; **f** LDH. Then the association between each SNP and clinical indicators were examined in the whole samples, early stage samples and late stage samples, respectively (Fig. 5)

We examined the difference of six clinical indicators between stages of lung adenocarcinoma (Fig. 4). There were significant differences in the six indicators between the early stage and late stage samples for both- or single-gender samples.

**Fig. 5:**
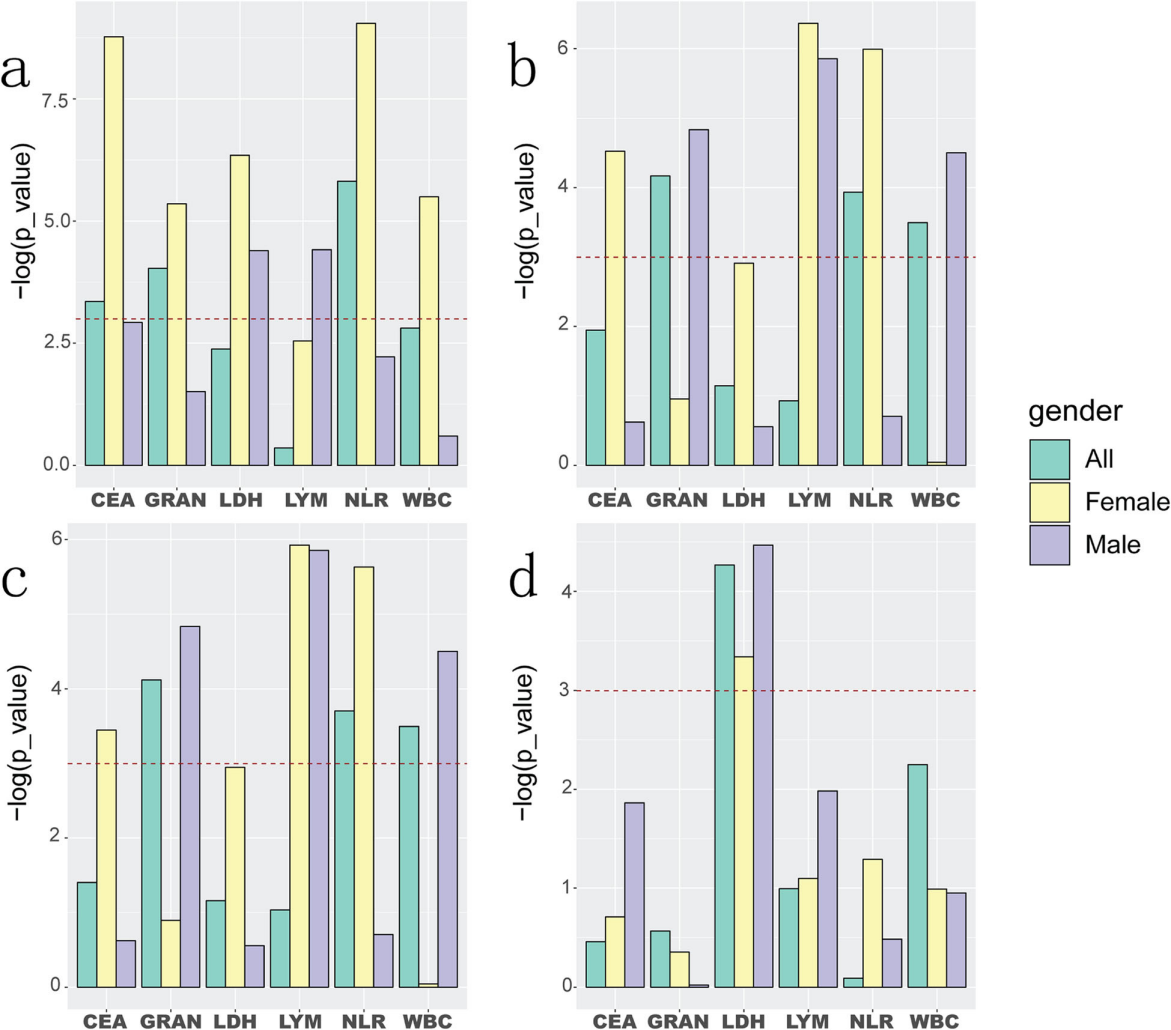
Association between SNPs and six indicators. **a** rs2227981; **b** rs2227982; **c** rs36084323; **d** rs7421861. The dotted red line indicates the significance threshold (*p*_value = 0.05)

It was found that rs7421861 was significantly correlated with LDH in both- or single-gender samples. Correlation between four SNPs and other clinical indicators varied in different gender groups. For all samples, there was a significant correlation between rs2227981 and three indicators: CEA, NLR, and GRAN. The genotyping of rs2227982 and rs36084323 were significantly correlated with NLR, GRAN, and WBC. For male samples alone, there was a significant correlation between rs2227981 and three indicators: CEA, LYM, and LDH. The genotyping of rs2227982 and rs36084323 were significantly correlated with LYM, GRAN, and WBC. For female samples alone, there was a significant correlation between rs2227981 and five indicators: CEA, NLR, GRAN, WBC, and LDH. The rs2227982 and rs36084323 were significantly correlated with CEA, NLR, and LYM.

The differences in gender between the correlations of these SNPs and clinical indicators may provide a reference for the clinical test results of patients.

### Classification efficiency evaluation of genes and clinical indicators

To evaluate the classification efficiency of PD-1, its target gene programmed death ligand 1 (PD-L1), CEA-encoding and LDH-encoding genes (other clinical indicators were based on cells without specific encoding genes), a Support Vector Machine (SVM) classifier was constructed for early stage and late stage samples, based on these genes. The gene expression data and clinical data were obtained from TCGA (https://cancergenome.nih.gov/). To assess the performance, AUC values were computed (Table 5). It can be seen that when PD-1 and PD-L1 genes were used, AUC is greater than 0.75 under different gender conditions, and was greater than that of LDH- and CEA-encoding genes.

**Table 5:** The classification efficiency of PD-1, PD-L1, CEA-encoding and LDH-encoding genes for both or single gender

Gene	AUC	Gender
PD-1_PD-L1	0.7904282	All
CEA_gene	0.6297458	All
LDH_gene	0.6192123	All
PD-1_PD-L1	0.767012	Male
CEA_gene	0.718195	Male
LDH_gene	0.715871	Male
PD-1_PD-L1	0.798156	Female
CEA_gene	0.720128	Female
LDH_gene	0.552606	Female

## Discussion

PD-1 and its ligand, PD-L1, were drug targets of lung adenocarcinoma [14]. In-depth study of their SNPs in the staging of lung adenocarcinoma may be helpful for the diagnosis and treatment of patients. Therefore, we selected 111 normal and 287 patients for the DNA extraction and genotyping, and the corresponding clinical indicators and stage information were analyzed. There was a certain correlation between four SNPs and the six clinical indicators. The significance of the correlation between clinical indicators and staging of lung adenocarcinoma of the two stages was higher than that of four stages. For two stages, PD-1 gene polymorphisms at three investigated positions and their haplotypes were associated with the risk and prognosis of lung adenocarcinoma in three genetic models (allele model, dominant model, and recessive model).

Based on SVM and LOOCV, CEA, LDH, and NLR could distinguish stages among the six clinical indicators. CEA was reported to be correlated with TNM stage and tumor invasion, and has become the marker of prognosis for lung adenocarcinomas [8]. The elevated NLR is an independent prognostic marker for poor survival at NSCLC, which has been used as a simple and useful tool to predict the prognosis of lung adenocarcinoma after radical pneumonectomy [11, 16]. LDH provided energy to tumor cells by converting acetone acid into lactic acid, and has been linked to prognosis of small cell lung cancer (SCLC) in a previous study [5]. PD-1 and PD-L1 could distinguish stages significantly, and their classification efficiency was higher than that of CEA and LDH related genes.

In view of the high classification efficiency of PD-1 and PD-L1 for staging and the significant correlation of their SNPs and haplotypes on them with staging, the polymorphism of PD-1 may be used as a marker for the staging of lung adenocarcinoma. Nevertheless, the data may be limited, for clinical data and SNP genotyping were obtained by our experiments, and the RNA-seq data were downloaded from TCGA, further evidence from other studies across different populations incorporating with the stage of cancers is required in order to confirm or refute the findings of this study.

## Conclusion

In summary, our study examined the PD-1 role in the disease process with patients collected prior to treatment, indicating that the PD-1 gene and the SNPs on it could be used as markers for distinguishing between early and late stages of lung adenocarcinoma in the Northeast Han population. Our investigation into the link between PD-1 polymorphisms and lung adenocarcinoma would help to provide guidance for the treatment and prognosis of lung adenocarcinoma.

## Data Availability

The data used and/or analyzed during the current study are available from the corresponding author upon a reasonable request.

## Abbreviations

CEA: Carcino-embryonic antigen
LDH: Lactate dehydrogenase
NLR: Neutrophil to Lymphocyte Ratio
NSCLC: Non-small cell lung cancer
PD-1: Programmed death 1
SCLC: Small cell lung cancer
SVM: Support Vector Machine
WBC: White blood cell
